# The impact of systemic lupus erythematosus on women's sexual functioning: A systematic review and meta-analysis

**DOI:** 10.1097/MD.0000000000007162

**Published:** 2017-07-07

**Authors:** Rulan Yin, Bin Xu, Lin Li, Ting Fu, Lijuan Zhang, Qiuxiang Zhang, Xia Li, Biyu Shen

**Affiliations:** a Department of Nursing, The Second Affiliated Hospital of Nantong University; b Nursing School of Nantong University, Nantong, Jiangsu, P.R. China.

**Keywords:** sexual function, systemic lupus erythematosus, women

## Abstract

**Background:**

A number of studies have reported the relationship between women's sexual problems and systemic lupus erythematosus (SLE). However, the results are contradictory. The objective of this paper was to explore the impact of SLE on women's sexual function.

**Methods:**

PubMed, Web of Science, CNKI Scholar, VIP and WanFang databases were searched up to April 2017. Studies evaluating the impact of SLE on women's sexual function with the use of Female Sexual Function Index (FSFI) scoring system were included. Statistical analyses were executed using version 5.0 Review Manager statistical software. Data were pooled using a fixed or random effects model according to heterogeneity.

**Results:**

A total of 2 identified studies matched the inclusion criteria, reporting on a total of 236 patients with SLE. No significant difference was observed between SLE patients and healthy controls on desire (*P* = .24; MD = −0.44 [−1.17, 0.29]), arousal (*P* = .12; MD = −0.39 [−0.89, 0.11]), lubrication (*P* = .17; MD = −0.53 [−1.28, 0.23]), orgasm (*P* = .27; MD = −0.27 [−0.75, 0.21]), satisfaction (*P* = .25; MD = −0.10 [−0.27, 0.07]) and pain (*P* = .17; MD = −0.50 [−1.22, 0.22]), except for total FSFI (*P* = .001; MD = −1.24 [−1.97, −0.50]).

**Conclusion:**

SLE has some influence on women’ sexual function. However, further studies of a larger population of female patients are required to further evaluate the mechanism by which SLE affects sexual function.

## 1 Introduction

Systemic lupus erythematosus (SLE) is an autoimmune chronic inflammatory disease of unknown etiology that can affect multiple organs and systems.^[1]^ Disease manifestations range from fatigue, skin rash, and arthralgias to central nervous system (CNS) involvement, arthritis, serositis, nephritis, pneumonitis, cardiac disease, and hematological problems.^[2,3]^ Due to its chronic nature, unpredictable course and widespread potential for harm, patients with SLE have a shorter life expectancy and reduced quality of life (QoL) compared to healthy sedentary population.^[4]^ Sexuality has been cited as an important part of the whole person, and sexual expression has been described as a crucial part of personal's self-identity.^[5]^ Sexual function plays a vital role in QoL and it is of particular significance for SLE patients because it occurs predominantly in women, especially young women, at a ratio of 9:1 (women:men).^[6]^

Previous studies have explored the association between women's sexual function and rheumatologic diseases, especially SLE.^[7–11]^ A previous study indicated that women with SLE had a lower desire, lubrication, and orgasm.^[7]^ A present study indicated that female SLE patients report lower sexual functioning, comparing with healthy women.^[9]^ However, another study showed that the prevalence of desire and orgasm in female patients with SLE was similar to that in healthy controls.^[8]^ Therefore, it is still controversial whether female SLE patients with sexual function is lower than normal women.

This paper aimed to investigate the impact of SLE on women's sexual function by performing a systematic review and meta-analysis of studies available in the literature.

## 2 Materials and methods

This systematic review and meta-analysis was performed adhering to the recommendations of the Preferred Reporting Items for Systematic Review and Meta-Analyses (PRISMA), and the Meta-analysis of Observational Studies in Epidemiology (MOOSE).^[12,13]^

### 2.1 Ethics statement

Because all the data collected and analyzed in this study are anonymous and do no potentially harm the patients, no effort is needed to seek consent from patients, ethical approval is unnecessary for the paper.

### 2.2 Literature search strategy

We conducted a systematic search on the English-language databases of PubMed and Web of Science, and Chinese databases of the CNKI Scholar, VIP and WanFang databases (from inception to April 2017) for investigations regarding SLE-related sexual function.

As shown below, the appropriate search strategy was used according to the language of the database. “Systemic lupus erythematosus” and “sexual function,” “sexual activity,” “sexual dysfunction,” or “sexual disorders” in title or abstract terms were used as key words for the English-language databases, and the Chinese translations of free text terms meaning SLE and sexual function were used for the Chinese databases. We also searched references of selected articles to identify additional reports.

### 2.3 Inclusion and exclusion criteria

Studies were included if the following criteria were met: Studies that evaluated the relationship between SLE and sexual function; the subjects enrolled fulfilled the American College of Rheumatology criteria for SLE; the subjects were female and up to 18 years old; and sexual function was assessed using the Female Sexual Function Index (FSFI)^[14]^ scoring system.

Authors of reports were contacted to clarify ambiguity for repeated studies of the same data. If the author was unavailable, we considered the first published study as original. A flowchart of this meta-analysis selection process was generated according to PRISMA requirements.

### 2.4 Data extraction

Two authors independently reviewed all full texts and extracted the data of the included studies. The reviewers would reach an agreement through discussion in the case of incomplete or unclear data. The quality of each study was evaluated independently using the Newcastle-Ottawa Scale.^[15]^ This scale uses a star system to evaluate nonrandomized studies regarding 3 criteria: patient selection, comparability of study groups, and outcome assessment. Studies achieving a rating of 6 stars or higher were considered to be of the highest quality.^[16]^

FSFI includes domains of sexual life, namely desire, arousal, lubrication, orgasm, satisfaction and pain preceding or during the sexual intercourse. If the articles were ambiguous or lacking outcomes, then contacted the author; if the author was not available, then extracted the relevant data through consensus.

### 2.5 Statistical analysis

Statistical analyses were executed using version 5.0 Review Manager statistical software. For continuous data, calculated the weighted mean differences (MDs), as well as 95% confidence interval (CI). Heterogeneity was assessed by *χ*^2^, *τ*^2^, and Higgins *I*^2^ tests, with significance set at *P* < .10. If there was no significant heterogeneity between studies, the fixed-effect model was used to combine these MDs to obtain an overall MD. Otherwise applied the random effects model. The overall effect was evaluated by *Z* score, and *P* < .05 was significant. Funnel plots and Egger test were adopted to evaluate publication bias when the number of studies included was more than 5.

## 3 Results

### 3.1 Study selection

The flowchart of this systematic review and meta-analysis selection process is presented in PRISMA Flow Diagram. A total of 2 studies, including 236 SLE women and 985 control women, were considered for this study.

### 3.2 Study characteristics

Two studies evaluated sexual function in female SLE patients.^[16]^Table 1 demonstrates the demographic and clinical characteristics of SLE patients and healthy controls. The 2 studies included were published as full text in 2011 and 2013, respectively. One study came from Spain, the other from Taiwan and the 2 studies were all published in English. The quality of the 2 studies was moderate.

**Table 1:**
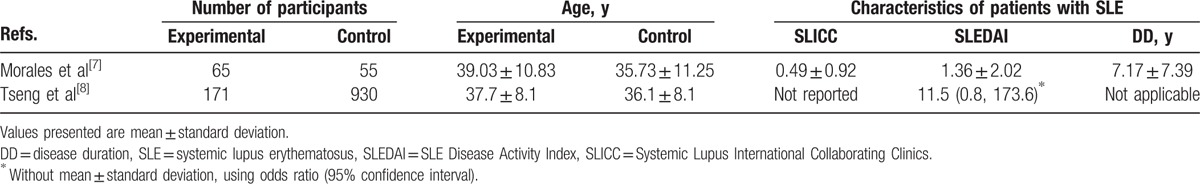
Clinical and demographic characteristics of patients with SLE and healthy controls.

### 3.3 Publication bias

Funnel plot analysis together with Egger tests were not performed to test publication bias since the number of studies included was less than 5.

### 3.4 Women's sexual function in SLE patients and healthy controls

**Figure 1:**
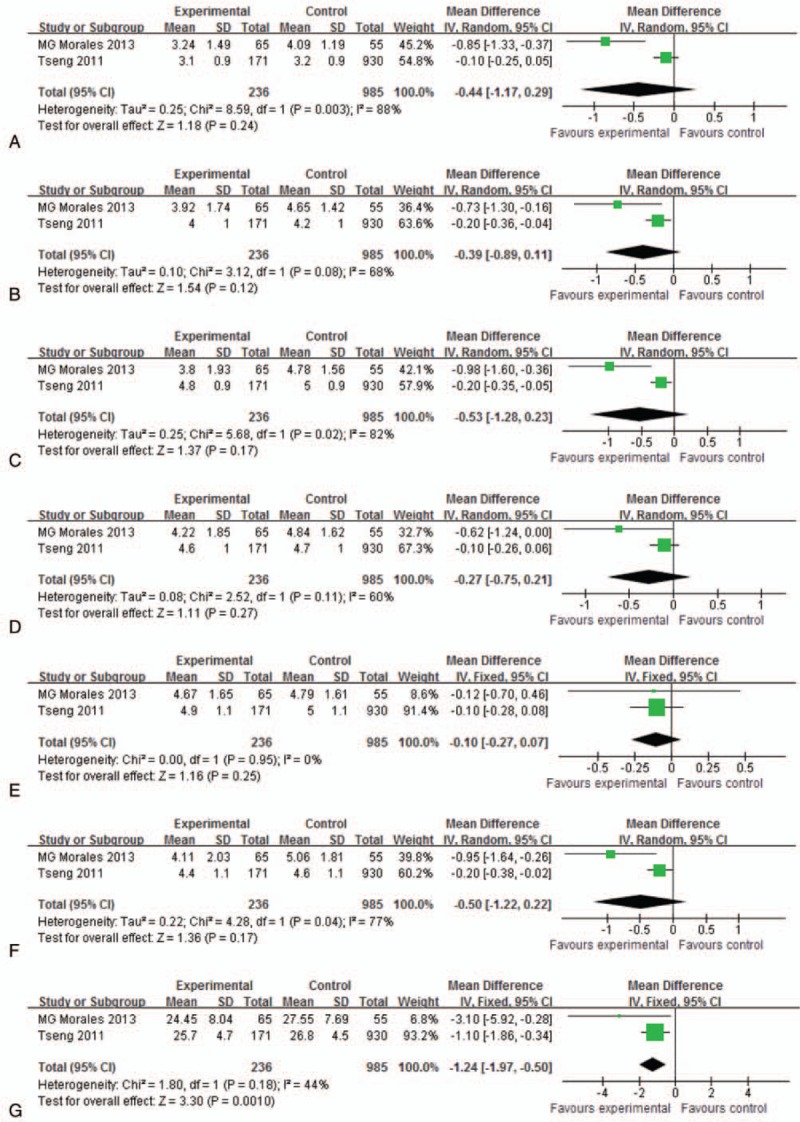
Female sexual function in SLE patients and controls. Forest plots of the Female Sexual Function Index (FSFI). (A) Desire, (B) arousal, (C) lubrication, (D) orgasm, (E) satisfaction, (F) pain, and (G) total FSFI. SLE = systemic lupus erythematosus.

The number of female SLE patients was from 65 to 171. Based on heterogeneity, the fixed effects model was applied to merge the domain of satisfaction and total FSFI, while the random effects model was used to combine the domains of desire, arousal, lubrication, orgasm, and pain. No significant difference was observed between SLE patients and healthy controls on desire (*P* = .24; MD = −0.44 [−1.17, 0.29]), arousal (*P* = .12; MD = −0.39 [−0.89, 0.11]), lubrication (*P* = .17; MD = −0.53 [−1.28, 0.23]), orgasm (*P* = .27; MD = −0.27 [−0.75, 0.21]), satisfaction (*P* = .25; MD = −0.10 [−0.27, 0.07]) and pain (*P* = .17; MD = −0.50 [−1.22, 0.22]), except for total FSFI (*P* = .001; MD = −1.24 [−1.97, −0.50]; Fig. 1; Table 2).

**Table 2:**
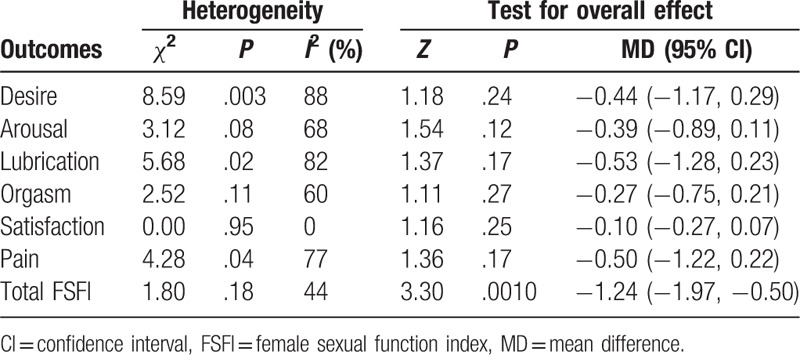
Results of meta-analysis for female sexual function.

## 4 Discussion

More and more evidence suggests that as an important part of QoL, sexual function is affected by SLE. As far as we know, the current meta-analysis which included 2 studies with a total of 236 participants is the first to quantitatively analyze the impact of SLE on women's sexual function. The results of this meta-analysis showed that female SLE patients have a lower total FSFI score, compared with healthy controls, which means that SLE had some influence on women's sexual function.

The pathogenesis of sexual problems in female SLE patients is still unclear. Previous studies have found that sexual function is significantly related to depression in female SLE patients^[6,7,9,17]^ and depression affects about 40% of SLE patients,^[18]^ so the clinical staff can improve patient's sexual function by improving the depressive state. Recent studies have shown that impairment of the normal structure and function of microglia, caused by autoimmune diseases, can result in depression and associated impairments in neuroplasticity and neurogenesis^[19]^ and interferon-α-induced activation of microglia was particularly relevant to depressive-like behavior.^[20]^ Additionally, a study in mice suggested that enriched environment-induced Adiponectin increase within the brain regulates microglia and brain macrophages phenotype and activation state, thus reducing neuroinflammation and depressive-like behaviors in mice.^[21]^

Previous study reported that SLE patients appear to report a lower sexual functioning than patients with others chronic illnesses.^[22]^ A review reported that sexual dysfunction in women with SLE is apparently most associated to vaginal discomfort or pain during intercourse.^[11]^ The risk factors of women's sexual problems in SLE patients are complicated and multifactorial. Sexual problems in female SLE patients may be associated with age, relationship status, weight concerns, premorbid sexual adjustment, and depression.^[9]^ Age, marital status, depression, and body image disturbance have been identified to be the most powerful predictors of impaired partner relationships, while body image to emotional distress, education, disease activity, and depression to be the most significant causes of impaired sexual function.^[6]^

Several shortcomings of the present study need to be addressed. First, the number of samples may not be large enough, although the amount of patients included in this study is greater than that in individual studies. Several studies were excluded in the present meta-analysis due to noncompliance with the FSFI. Second, there was major heterogeneity in the study of sexual function in female patients with SLE. It is assumed that the difference in disease activity and patients’ characteristics is responsible for heterogeneity. For that reason, all the conclusions need to be carefully considered.

## 5 Conclusion

There is a relationship between women's sexual function and SLE. Female patients with SLE appear to have lower sexual function than healthy people. It is of the essence to diagnose sexual dysfunction for female SLE patients with sexual problems as soon as possible. Hence, clinicians should realize the impact of SLE on women’ sexual health, and keep a watchful eye on all aspects of life, not just physical function and disease activity.

## References

[1] Bogdanovic G Stojanovich L Djokovic A Physical activity program is helpful for improving quality of life in patients with systemic lupus erythematosus. Tohoku J Exp Med 2015;237:193–9. 26490344 10.1620/tjem.237.193

[2] Oomatia A Fang H Petri M Peripheral neuropathies in systemic lupus erythematosus: clinical features, disease associations, and immunologic characteristics evaluated over a twenty-five-year study period. Arthritis Rheumatol 2014;66:1000–9. 24757151 10.1002/art.38302

[3] Ermann J Bermas BL The biology behind the new therapies for SLE. Int J Clin Pract 2007;61:2113–9. 17997810 10.1111/j.1742-1241.2007.01528.x

[4] Tench C Bentley D Vleck V Aerobic fitness, fatigue, and physical disability in systemic lupus erythematosus. J Rheumatol 2002;29:474–81. 11908559

[5] Hill J Effects of rheumatoid arthritis on sexual activity and relationships. Rheumatology 2003;42:280–6. 12595623 10.1093/rheumatology/keg079

[6] Shen B He Y Chen H Body image disturbances have impact on the sexual problems in Chinese systemic lupus erythematosus patients. J Immunol Res 2015;2015:1–6. 10.1155/2015/204513 PMC4451262 26090484

[7] Morales MG Rubio JC Peralta-Ramirez M Impaired sexual function in women with systemic lupus erythematosus: a cross-sectional study. Lupus 2013;22:987–95. 23963430 10.1177/0961203313500370

[8] Tseng JC Lu LY Hu JC The impact of systemic lupus erythematosus on women's sexual functioning. J Sex Med 2011;8:3389–97. 21951616 10.1111/j.1743-6109.2011.02464.x

[9] Curry SL Levine SB Corty E The impact of systemic lupus erythematosus on women's sexual functioning. J Rheumatol 1994;21:2254–60. 7699626

[10] Østensen M New insights into sexual functioning and fertility in rheumatic diseases. Best Pract Res Clin Rheumatol 2004;18:219–32. 15121041 10.1016/j.berh.2004.01.002

[11] Tristano AG The impact of rheumatic diseases on sexual function. Rheumatol Int 2009;29:853–60. 19152092 10.1007/s00296-009-0850-6

[12] Stroup DF Berlin JA Morton SC Meta-analysis of observational studies in epidemiology: a proposal for reporting. Meta-analysis Of Observational Studies in Epidemiology (MOOSE) group. JAMA 2000;283:2008–12. 10789670 10.1001/jama.283.15.2008

[13] Moher D Liberati A Tetzlaff J Preferred reporting items for systematic reviews and meta-analyses: the PRISMA statement. Ann Intern Med 2009;151:264–9. W64. 19622511 10.7326/0003-4819-151-4-200908180-00135

[14] Rosen R Brown C Heiman J The Female Sexual Function Index (FSFI): a multidimensional self-report instrument for the assessment of female sexual function. J Sex Marital Ther 2000;26:191–208. 10782451 10.1080/009262300278597

[15] Stang A Critical evaluation of the Newcastle-Ottawa scale for the assessment of the quality of nonrandomized studies in meta-analyses. Eur J Epidemiol 2010;25:603–5. 20652370 10.1007/s10654-010-9491-z

[16] Mahid SS Minor KS Soto RE Smoking and inflammatory bowel disease: a meta-analysis. Mayo Clin Proc 2006;81:1462–71. 17120402 10.4065/81.11.1462

[17] Anyfanti P Pyrpasopoulou A Triantafyllou G Association between mental health disorders and sexual dysfunction in patients suffering from rheumatic diseases. J Sex Med 2014;2653–60. 25124339 10.1111/jsm.12672

[18] Stock AD Wen J Doerner J Neuropsychiatric systemic lupus erythematosus persists despite attenuation of systemic disease in MRL/lpr mice. J Neuroinflammation 2015;12:205. 26546449 10.1186/s12974-015-0423-4 PMC4636802

[19] Yirmiya R Rimmerman N Reshef R Depression as a microglial disease. Trends Neurosci 2015;38:637–58. 26442697 10.1016/j.tins.2015.08.001

[20] Wachholz S Eßlinger M Plümper J Microglia activation is associated with IFN-alpha induced depressive-like behavior. Brain Behav Immun 2016;55:105–13. 26408795 10.1016/j.bbi.2015.09.016

[21] Chabry J Nicolas S Cazareth J Enriched environment decreases microglia and brain macrophages inflammatory phenotypes through adiponectin-dependent mechanisms: relevance to depressive-like behavior. Brain Behav Immun 2015;50:275–87. 26209808 10.1016/j.bbi.2015.07.018

[22] Daleboudt GMN Broadbent E McQueen F The impact of illness perceptions on sexual functioning in patients with systemic lupus erythematosus. J Psychosom Res 2013;74:260–4. 23438719 10.1016/j.jpsychores.2012.11.004

